# Ectopic Expression of Multiple Chrysanthemum (*Chrysanthemum × morifolium*) R2R3-MYB Transcription Factor Genes Regulates Anthocyanin Accumulation in Tobacco

**DOI:** 10.3390/genes10100777

**Published:** 2019-10-04

**Authors:** Yan Hong, Mengling Li, Silan Dai

**Affiliations:** School of Landscape Architecture, Beijing Forestry University, No. 35 Tsinghua East Road, 100083 Beijing, China; hongy@bjfu.edu.cn (Y.H.); lmling1002@sina.com (M.L.)

**Keywords:** *Chrysanthemum × morifolium*, R2R3-MYB, transcription factor, anthocyanin, biosynthesis

## Abstract

The generation of chrysanthemum (*Chrysanthemum × morifolium*) flower color is mainly attributed to the accumulation of anthocyanins. In the anthocyanin biosynthetic pathway in chrysanthemum, although all of the structural genes have been cloned, the regulatory function of R2R3-MYB transcription factor (TF) genes, which play a crucial role in determining anthocyanin accumulation in many ornamental crops, still remains unclear. In our previous study, four light-induced R2R3-MYB TF genes in chrysanthemum were identified using transcriptomic sequencing. In the present study, we further investigated the regulatory functions of these genes via phylogenetic and alignment analyses of amino acid sequences, which were subsequently verified by phenotypic, pigmental, and structural gene expression analyses in transgenic tobacco lines. As revealed by phylogenetic and alignment analyses, *CmMYB4* and *CmMYB5* were phenylpropanoid and flavonoid repressor R2R3-MYB genes, respectively, while *CmMYB6* was an activator of anthocyanin biosynthesis, and *CmMYB7* was involved in regulating flavonol biosynthesis. Compared with wild-type plants, the relative anthocyanin contents in the *35S:CmMYB4* and *35S:CmMYB5* tobacco lines significantly decreased (*p* < 0.05), while for *35S:CmMYB6* and *35S:CmMYB7*, the opposite result was obtained. Both in the *35S:CmMYB4* and *35S:CmMYB5* lines, the relative expression of several anthocyanin biosynthetic genes in tobacco was significantly downregulated (*p* < 0.05); on the contrary, several genes were upregulated in the *35S:CmMYB6* and *35S:CmMYB7* lines. These results indicate that *CmMYB4* and *CmMYB5* negatively regulate anthocyanin biosynthesis in chrysanthemum, while *CmMYB6* and *CmMYB7* play a positive role, which will aid in understanding the complex mechanism regulating floral pigmentation in chrysanthemum and the functional divergence of the R2R3-MYB gene family in higher plants.

## 1. Introduction

Ornamental plants with novel colors and coloration patterns are of great commercial value in the floricultural industry, as they are among the most important aesthetic characters for consumers [1]. Pigmentation in flowers is mainly attributed to anthocyanins, a type of flavonoid; the major pigments imparting a great variety of colors from light yellow, red, dark red, magenta, purple, to blue in ornamental plants have been investigated extensively [2,3,4].

Based on their DNA binding domains, MYB transcription factors (TFs) are generally classified into four main types, namely, R2, R2R3, R3, and R4 types, of which R2R3-MYBs are closely related to anthocyanin biosynthesis [5]. Anthocyanin biosynthesis is primarily controlled by a complex consisting of R2R3-MYB, basic helix–loop–helix (bHLH), and WD40 proteins that acts as the master regulator to coordinate the transcription of anthocyanin biosynthetic genes. In particular, among the three different types of proteins, R2R3-MYBs have been shown to play crucial roles in anthocyanin accumulation in many plants, including model and horticultural crops, because of their specificity in regulating the expression of structural genes involved in the anthocyanin biosynthetic pathway [2,6,7,8,9,10,11,12,13,14]. For example, Zhang et al. 2019 identified three novel R2R3-MYB members of anthocyanin regulators in the genome of the purple flowering *Petunia inflate*, which were specifically expressed with the development of various tissues; transcript levels of many anthocyanin biosynthetic genes, such as *CHSaI* (chalcone synthase), *CHSj*, *F3H* (flavanone 3-hydroxylase), *F3′5′H* (flavonoid-3′,5′-hydroxylase), *DFR* (dihydroflavonol 4-reductase), and *ANS* (anthocyanin synthase), were increased in the overexpression lines of *P. hybrida* line M1 compared to untransformed controls [15]. In the anthocyanin biosynthetic pathway, structural genes *AtCHS* (*Arabidopsis thaliana*), *AtCHI* (chalcone isomerase), *AtF3H*, *AtF3′H* (flavonoid-3′-hydroxylase), *AtDFR*, and *AtANS* were enhanced in the *FtMYB15* (*Fagopyrum tataricum*)-transgenic Arabidopsis lines [16]. Similarly, the heterologous expression of *OjMYB1* (*Oenanthe javanica*) in Arabidopsis enhanced the anthocyanin content and upregulated the expression levels of several structural genes related to anthocyanin biosynthesis [17]. In addition to the proteins involved in activating the anthocyanin biosynthetic genes, distinct R2R3-MYB factors have also been identified as repressors of anthocyanin biosynthesis, which actively repress target genes mediated by motifs in their C-terminus [18,19]. FaMYB1, an R2R3-MYB from *Fragaria × ananassa*, was shown to interact with the bHLH factors AN1 and JAF13 from *P. hybrida*; ectopic expression of FaMYB1 in tobacco (*Nicotiana tabacum*) repressed the transcription levels of ANS and inhibited the biosynthesis and accumulation of anthocyanins in flowers [20]. Therefore, investigating the specific regulation of R2R3-MYBs towards the biosynthesis and accumulation of anthocyanins aids in clearly understanding the complex mechanism of floral pigmentation in higher plants.

Chrysanthemum (*Chrysanthemum × morifolium*) is a world-renowned ornamental crop with rich flower colors, whose yield and output values make it outstanding in the global flower industry [21]. Interestingly, only one anthocyanin biosynthetic pathway, the cyanidin metabolic pathway, exists in the chrysanthemum [22,23], making it an ideal model for studies of anthocyanin biosynthesis. However, to date, the R2R3-MYB studies in chrysanthemum have mainly focused on stress tolerance, e.g., drought, salinity [24], and aphid [25] tolerance, while R2R3-MYB members regulate anthocyanin biosynthesis, and their regulatory functions are rarely reported, resulting in less understanding of the pigmentation mechanism of chrysanthemum flowers and representing an obstacle to molecular breeding for novel flower colors.

In a previous study, the authors identified four light-induced R2R3-MYB TF genes in the chrysanthemum using transcriptomic sequencing [26], namely *CmMYB4*, *CmMYB5*, *CmMYB6*, and *CmMYB7*, but their regulatory functions were not revealed. Here, we further investigated their functions via alignment and phylogenetic analyses of amino acid sequences, which were subsequently verified by phenotypic, pigmental, and structural gene expression analyses in transgenic tobacco lines. This study will be helpful for improving functional genomic studies in chrysanthemums and will further our understanding of the molecular mechanisms of anthocyanin biosynthesis and accumulation.

## 2. Materials and Methods

### 2.1. Plant Materials

The chrysanthemum cultivar ‘Purple Reagan’ and tobacco line “NC89” were selected as the experimental materials, which were planted in pots containing compost mix (peat:vermiculite = 1:1) in September 2018 and maintained in an artificial climate chamber (intensity of illumination = 50 ± 5 μmol m^−2^ s^−1^, temperature = 20 ± 2 °C, and relative humidity = 60%) of Beijing Forestry University, Beijing, China. The photoperiod for chrysanthemum seedlings was 16 h day/8 h night before they grew to 14 leaves, which were transferred to short-day conditions (8 h day/16 h night) thereafter until flowering after 43 days of growth. The photoperiod for tobacco seedlings was 8 h day/16 h night until flowering.

Ray florets of chrysanthemum seedlings and corollas of tobacco seedlings at the full-bloom stage were collected in October 2018 and were subsequently rapidly frozen in liquid nitrogen and kept at −80 °C for further analyses.

### 2.2. Isolation of TF Genes

Total RNA was extracted from the ray florets of chrysanthemum seedlings using the Quick RNA Isolation Kit (Huayueyang Biotechnology Co. Ltd., Beijing, China), and first-strand cDNA was synthesized by the M-MLV Reverse Transcription Kit (Promega, Madison, WI, USA) according to the manufacturer’s protocol. All of the CmMYB sequences were obtained from the chrysanthemum RNA-Seq database, which was constructed using the ray florets of the capitulum during different developmental stages and light conditions in our previous study [26]. The open reading frames of *CmMYB4*, *CmMYB5*, *CmMYB6*, and *CmMYB7* were acquired by PCR amplification from the cDNA with specific primers (Table 1). Amplification conditions were previously described [26].

### 2.3. Phylogenetic Clustering and Amino Acid Sequence Alignment

A phylogenetic tree was constructed according to the neighbor-joining statistical method. Tree nodes were evaluated using the bootstrap method for 1000 replicates, and branches corresponding to partitions reproduced in less than 50% of the bootstrap replicates were condensed into single branches. Evolutionary distances were computed using the *p*-distance method and expressed in units of amino acid differences per site. All positions containing gaps and missing data were eliminated prior to construction of the phylogenetic trees. Amino acid sequences of the R2R3-MYB proteins in the chrysanthemum and other species were aligned in MEGA software (version 6.0; Pennsylvania State University, PA, USA). The conserved motifs of each R2R3-MYB subgroup (SG) were predicted using MEME (version 5.0.5; National Institutes of Health, Bethesda, MA, USA). The GenBank accession numbers are shown in Supplementary Table S1. Multiple sequence alignment was performed using DNAMAN software (version 7.0; Lynnon Biosoft, San Ramon, CA, USA). 

### 2.4. Expression Vector Construction and Tobacco Transformation

The cDNA sequences of *CmMYB4*, *CmMYB5*, *CmMYB6*, and *CmMYB7* were amplified using specific primers containing *Bam*HI and *Eco*RI restriction sites (Table 1). The products were each cloned into the pGEM-T vector (Promega, Madison, WI, USA) and ascertained by Sanger sequencing. The inserts were ligated into the modified pBI121 vector to obtain recombinant vectors after digestion with restriction enzymes. The *35S:CmMYB4*, *35S:CmMYB5*, *35S:CmMYB6,* and *35S:CmMYB7* vectors were subsequently transformed into *Agrobacterium tumefaciens* strain GV3101.

Tobacco ‘NC89’ plants were transformed using an *A. tumefaciens*-mediated leaf disc procedure [27] and were selected using 100 mg/L kanamycin. The kanamycin-resistant T_0_ generation of tobacco lines was transferred to a growth chamber after rooting and acclimatization to harvest T_1_ generation seeds. Subsequently, T_2_ generation transgenic seeds were harvested for further assays [28].

### 2.5. Anthocyanin Component Analysis

The extraction of anthocyanins was performed according to previous methods [23]. Briefly, 0.25 g of fresh tobacco corolla was collected and ground into powder under liquid nitrogen and then extracted with 1 mL of 0.1% HCl-methanol in the dark at 4 °C for 24 h, with vortexing every 6 h. Then, the supernatants were collected and passed through 0.22 μm-reinforced nylon membrane filters (Shanghai ANPEL, Shanghai, China) before high-performance liquid chromatography (HPLC) analysis. Three replicates were performed for each treatment.

HPLC analysis was performed using an Agilent 1100 Liquid Chromatograph (Agilent Technologies Inc., La Jolla, CA, USA). The column temperature was 35 °C, and the injection volume was 10 μL. The analytical column was eluted with mobile phase A (formic acid:water = 1:9, v:v) and mobile phase B (acetonitrile:methanol = 85:15, v:v) at a rate of 0.8 mL min^−1^. The elution procedure was as follows: 0 min, 10% B; 10 min, 20% B; 12 min, 50% B; 14 min, 10% B; 15 min, 10% B. The resulting chromatograms were read at 515 nm for anthocyanins.

### 2.6. qRT-PCR Analysis

The expression abundance of anthocyanin biosynthesis structural genes in transgenic tobacco corolla was quantified by qRT-PCR. PCR reactions were conducted using a Mini Opticon Real-time PCR System (Bio-Rad, Hercules, CA, USA) based on SYBR Premix ExTaq (TaKaRa, Shiga, Japan) with three independent transgenic lines as replicates. The *Ntα-Tub1* (GenBank accession number: AJ421411) gene, which was constantly expressed in RNA-Seq data sets, was used as an endogenous control to calculate the relative expression levels of target genes. Primer sequences are listed in Table 1.

## 3. Results

### 3.1. Phylogenetic and Sequence Alignment Analyses

**Figure 1:**
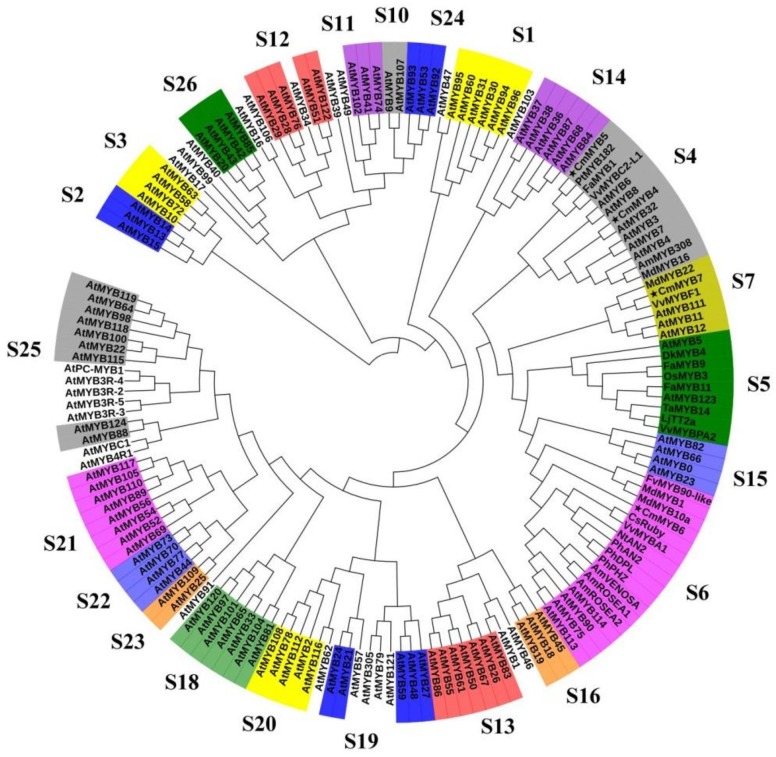
Phylogenetic relationships of MYBs in other plants compared to the four MYBs from the chrysanthemum. Full-length amino acid sequences of MYBs from all species were first aligned using ClustalW in MEGA. The phylogenetic tree was constructed according to the neighbor-joining method. Branches corresponding to partitions reproduced in less than 50% of the bootstrap replicates were collapsed. The evolutionary distances were computed using the *p*-distance method. All ambiguous positions were removed for each sequence pair. Stars represent the four CmMYB proteins. S represents subgroup. The GenBank accession numbers of each species are shown in Supplementary Table S1.

Phylogenetic analysis showed that all four CmMYBs were clustered with experimentally characterized MYBs in other plants. For example, CmMYB4 was closely related to general phenylpropanoid repressor MYBs, such as AmMYB308 (*Antirrhinum majus*), AtMYB4, AtMYB32, and AtMYB6, while CmMYB5 was similar to flavonoid MYB repressors, e.g., FaMYB1, VvMYBC2-L1 (*Vitis vinifera*), and PtMYB182 (*Populus tremula*). CmMYB6 was closely related to anthocyanin MYB activators, including VvMYBA1, CsRuby (*Citrus sinensis*), MdMYB10a (*Malus domestica*), PhAN2, AtPAP1, etc. Finally, CmMYB7 was clustered into the group related to flavonol biosynthesis together with, e.g., VvMYBF1, MdMYB22, AtMYB11, AtMYB12, and AtMYB111 (Figure 1).

**Figure 2:**
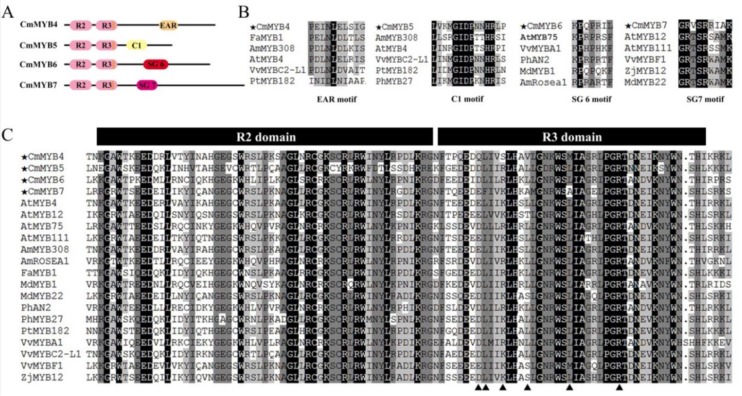
Diagram and alignment of R2R3-MYBs related to flavonoid biosynthesis regulation in other plants compared to the four MYBs from the chrysanthemum. (**A**) Structural diagram of the four CmMYB transcription factors. R2 and R3 domains are marked by pink; orange bars in the R3 domain represent bHLH-binding sites; dark yellow, light yellow, red, and purple indicate EAR, C1, SG6, and SG7 motifs, respectively. (**B**) Amino acid sequence alignment of EAR, C1, SG6, and SG7 motifs contained in the four CmMYB transcription factors and in other plants. (**C**) Amino acid sequence alignment of the R2 and R3 domains contained in the four CmMYB transcription factors and in other plants. The GenBank accession numbers of each species are shown in Supplementary Table S1. EAR, element-binding factor-associated amphiphilic repression; SG, subgroup.

Sequence alignment revealed that all four CmMYB proteins contained highly conserved R2 and R3 domains. A characteristic identifier of dicot anthocyanin-promoting MYBs, ANDV, was also found in the R3 domain of CmMYB6. Moreover, a bHLH-binding motif, namely [DE]Lx_2_[RK]x_3_Lx_6_Lx_3_R, was included in the R3 domain of all proteins, indicating that these MYBs might have protein–protein interactions with R/B-like bHLHs (Figure 2).

Although the N-terminus of the four CmMYB TFs was conserved, high diversity was detected in their C-terminus. CmMYB4 contained a C2 motif (LxLxL), which is also known as an ethylene-responsive element binding factor-associated amphiphilic repression (EAR) motif, on its C-terminal domain. This motif has been approved as a conserved repression domain in the R2R3-MYB SG4, although instead of the common LNLDL sequence, a conservative substitution INLEL was found. CmMYB5 contained a C1 motif (LlsrGIDPxT/SHRxI/L), which was also a conserved element in R2R3-MYB SG4. The SG6 ([K/R]Pxxx[K/T][F/Y]) and SG7 ([K/R][R/x][R/K]xGRT[S/x][R/G]xx[M/x]K) motifs were detected in the C-terminus of CmMYB6 and CmMYB7, respectively. Moreover, a conserved sequence containing GRVSRRIAK was found in SG7 motif instead of the most common GRTSRWAMK sequence (Figure 2).

Based on the above results, we speculated that in the chrysanthemum, *CmMYB4* and *CmMYB5* are phenylpropanoid and flavonoid repressor genes, respectively, while *CmMYB6* is an activator for anthocyanin biosynthesis; *CmMYB7* is involved in regulating flavonol biosynthesis.

### 3.2. Pigmental and Phenotypic Analyses of Transgenic Tobacco Lines

**Figure 3:**
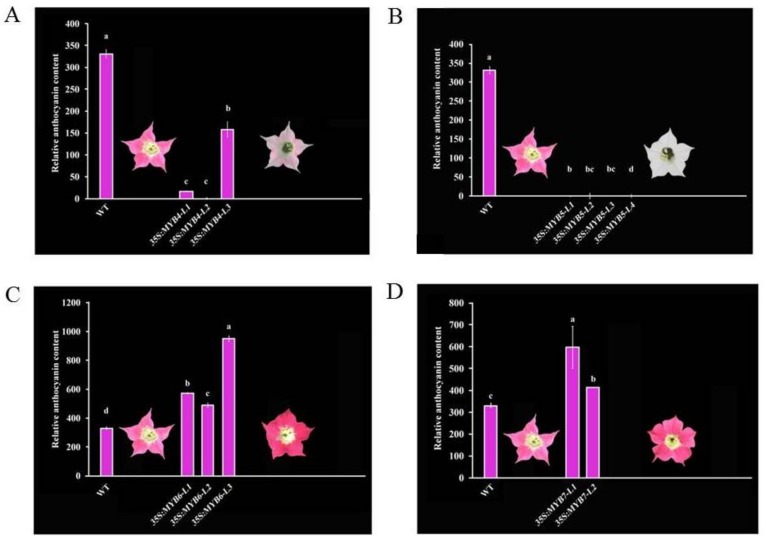
Pigmental and phenotypic changes in the (**A**) *35S:CmMYB4,* (**B**) *35S:CmMYB5,* (**C**) *35S:CmMYB6*, and (**D**) *35S:CmMYB7* tobacco lines. The results are presented as the mean ± SD of three replicates. Different letters represent significant differences at *p* < 0.05 between different samples. L1, L2, L3, and L4 indicate independent transgenic tobacco lines obtained in the present study. WT, wild type.

To further validate the regulatory functions of CmMYBs for anthocyanin accumulation, the changes in relative anthocyanin content and flower phenotype were investigated in the transgenic tobacco lines. As a result, compared with the wild-type plants (control group), the relative anthocyanin content in the *35S:CmMYB4* (Figure 3A) and *35S:CmMYB5* (Figure 3B) lines significantly decreased (*p* < 0.05), resulting in the fading of tobacco corolla color; in particular, the tobacco flowers were almost changed to white for the latter. On the contrary, in the *35S:CmMYB6* (Figure 3C) and *35S:CmMYB7* (Figure 3D) lines, the relative anthocyanin content in tobacco corolla significantly increased, accompanied by the deepening of flower color. These results indicated the repressive function of CmMYB4 and CmMYB5 and the activating role of CmMYB6 and CmMYB7 for anthocyanin accumulation in the chrysanthemum.

### 3.3. Expression of Anthocyanin Biosynthetic Genes in Transgenic Tobacco Lines

**Figure 4:**
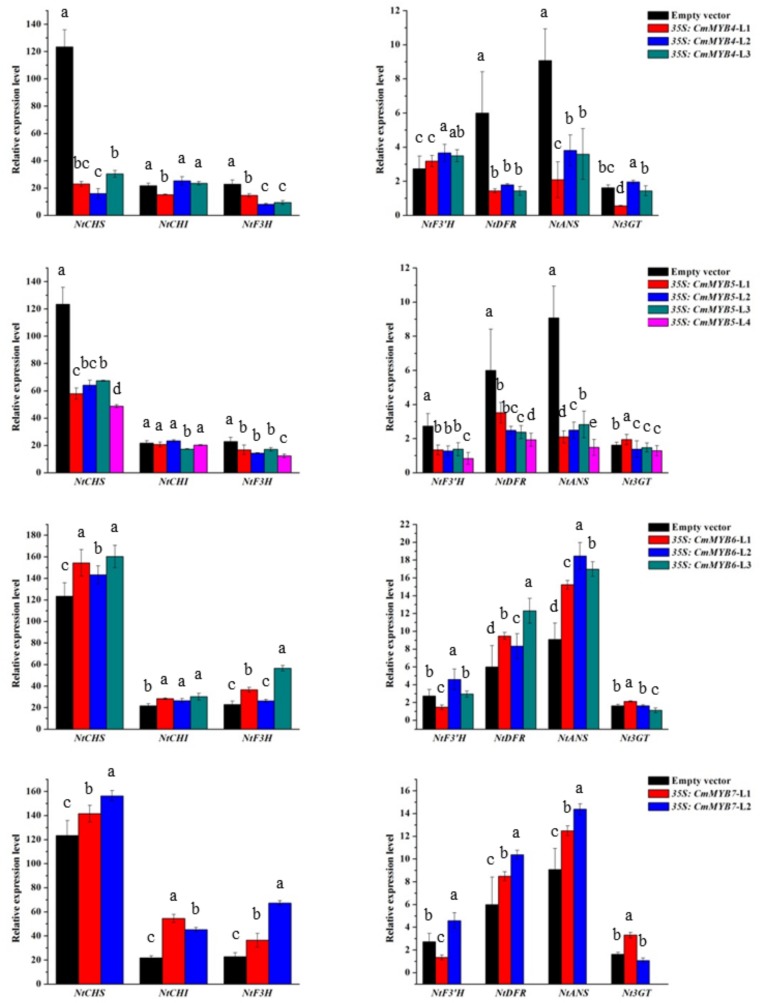
The relative expression levels of seven anthocyanin biosynthetic genes in the *35S: CmMYBs* tobacco lines. The results are presented as the mean ± SD of three replicates. Different letters represent significant differences at *p* < 0.05 between different samples. L1, L2, L3, and L4 indicate independent transgenic tobacco lines obtained in the present study.

Ectopic gene expression analysis in tobacco lines showed that the transcriptional levels of two early genes, *NtCHS* and *NtF3H*, together with two late genes, *NtDFR* and *NtANS*, were significantly downregulated in both of the *35S:CmMYB4* and *35S:CmMYB5* lines. Additionally, *NtF3′H* was downregulated in the *35S:CmMYB5* lines (*p* < 0.05). On the contrary, the relative expression levels of all three early genes, *NtCHS*, *NtCHI,* and *NtF3H*, together with two late genes, *NtDFR* and *NtANS*, were significantly upregulated in both of the *35S:CmMYB6* and *35S:CmMYB7* lines (*p* < 0.05). No obvious expression pattern was found for *Nt3GT* (Figure 4).

From the above results, we can conclude that *CmMYB4* and *CmMYB5* repress the anthocyanin accumulation in chrysanthemums via downregulation of the transcriptional levels of anthocyanin biosynthetic genes, while *CmMYB6* and *CmMYB7* play a positive role, although the target structural genes might be different.

## 4. Discussion

There is increasing interest in the modification of flower colors. Metabolic genetic engineering via anthocyanin regulatory MYBs can change cell metabolism by altering its pathway enzyme(s) or regulatory protein(s) using recombinant DNA technology; thus, metabolic genetic engineering of MYBs has generated diverse colors and color patterns in many ornamental plants [4,29]. Chrysanthemum contains thousands of cultivars which are classed into nine groups based on different flower colors that are mainly influenced by anthocyanin contents [30]. However, because the pigmentation mechanism of chrysanthemum ray florets is unclear at present, particularly the indistinct regulatory functions of R2R3-MYBs for anthocyanin biosynthesis and accumulation, genetic engineering of novel flower colors in chrysanthemums is rarely reported. In this study, the identities and regulatory functions of four R2R3-MYB TF genes in the chrysanthemum were investigated by multiple lines of evidence, which will aid in understanding the complex mechanism regulating floral pigmentation in the chrysanthemum and will pave the way forward for their genetic engineering.

### 4.1. All Four CmMYB TFs Are R2R3-MYBs with Diverse Functional Motifs in the C-Terminus

It is widely accepted that the function of R2R3-MYB TFs of the same SG is highly conserved amongst angiosperms [31,32]. In the present study, all four CmMYB proteins were approved as R2R3-MYB because, similar to well-known R2R3-MYBs in other plants such as Arabidopsis, apples, petunias, grapevine, and maize (*Zea mays*), highly conserved R2 and R3 domains were detected in their N-termini, indicating that the four CmMYB TFs share similar functions with orthologs in their corresponding SGs.

The R2R3-MYB repressors contain domains in their C-terminus required for their repressive activity [18,19], such as the C2 region with the core EAR motif, which actively represses transcription [33]. In particular, members of SG4 R2R3-MYB proteins contain the C2 motif, which represses flavonoid biosynthetic genes [34,35,36,37]. In tobacco ERF3 (NtERF3), where the EAR motif was first characterized, deletion mutations within the EAR motif eliminated its ability to repress transcription [33]. Although the mechanism of repression is still unclear, some reports show that the EAR motif is important for interaction with histone modification proteins [38]. Arabidopsis EAR domain-containing proteins, e.g., AtMYB4, AtMYB7, and AtMYB32, show the DLNxxP or LxLxL sequence conservation pattern within their core sites [39]. Nevertheless, conservative substitutions were also shown in many plants; for example, PhMYB27 has a DLNSPP signature [40], while PtMYB181, PtMYB182, and AtMYBL2 contain a slightly modified EAR motif, consisting of INLDL, IxIxL, and DLNIGL, respectively [41,42]. Moreover, it was also reported that the co-repressor TOPLESS, a bridge protein linking TFs and the HDAC complex, interacts with the auxin repressor IAA12 and the JAZ repressor NINJA via EAR motif in Arabidopsis [43,44], which modifies chromatin structure and thus changes transcriptional activity [45]. In the present study, a C2 motif with a conservative substitution, INLEL, in the C-terminal domain of CmMYB4—a regulator for general phenylpropanoid as revealed by phylogenetic analysis—was also detected, suggesting a similar repressive function of this SG4 R2R3-MYB. To our best knowledge, the conservative substitution, INLEL, in the EAR motif has never been reported amongst higher plants; whether it is specific in chrysanthemums needs further verification using, for example, pull-down assays and proteomic approaches. The putative interactions between TOPLESS and EAR motif in the chrysanthemum still remain to be disclosed.

The C1 motif, also called the GIDP motif, has been reported as another diagnostic motif among SG4 R2R3-MYBs [20,34,42,46]. CmMYB5 is another putative SG4 repressor MYB that is phylogenetically closed to well-known flavonoid repressors in other plants containing the same motif.

Within the variable C-terminal region, the conserved SG6 motif has been widely identified as defining the anthocyanin-related proteins [47,48,49,50,51]. At an early stage, the SG6 motif was identified as KPRPR[S/T]F in Arabidopsis [47] and thereafter modified to [K/R]P[Q/R]P[Q/R] based on a study in Asiatic lily [48]. R2R3-MYB proteins of SG7 have been shown to regulate the generation of flavonols in different eudicot plants and in the monocot plant maize [47,52]. The combination of the SG7 motif, [K/R][R/x][R/K]xGRT[S/x][R/G]xx[M/x]K, with the R2R3 domain is a useful tool for the initial identification of R2R3-MYB proteins that regulate flavonol biosynthesis [52]. In the present study, CmMYB6 and CmMYB7 were characterized by the SG6 and SG7 motifs, respectively; the amino acid sequences of these two MYBs showed high homology with other orthologs containing the SG6 or SG7 motifs from plants in their corresponding SGs. Therefore, CmMYB6 is a putative regulator of anthocyanin biosynthesis, while CmMYB7 might be responsible for the regulation of flavonoids.

### 4.2. CmMYB4 and CmMYB5 Are Anthocyanin Repressors, while CmMYB6 and CmMYB7 Are Activators

The biosynthesis of anthocyanins and other flavonoids is well characterized, and there is an increasing understanding of the mechanisms that regulate their production [4,12]. Consequently, the regulatory networks governing anthocyanin accumulation have become important models for understanding how metabolic pathways are controlled in plants and how patterned gene expression can occur [7].

Generally, anthocyanins (cyanidin, pelargonidin, and delphinidin derivatives) are synthesized via three main branches of the biosynthetic pathway. Transcriptional regulation of the structural genes involved in the three branches, such as *CHS*, *CHI*, *F3H*, *F3′H*, *F3′5′H*, *DFR*, *ANS*, and *3GT* (*UFGT*; flavonoid 3-O-glucosyltransferase), leads to the generation of diverse flavonoid pigments in plant species [53]. Structural homology among MYB proteins from different plant species might point to a general similarity in the pathways regulated by them and in the type of regulation—activation or repression [20].

**Figure 5:**
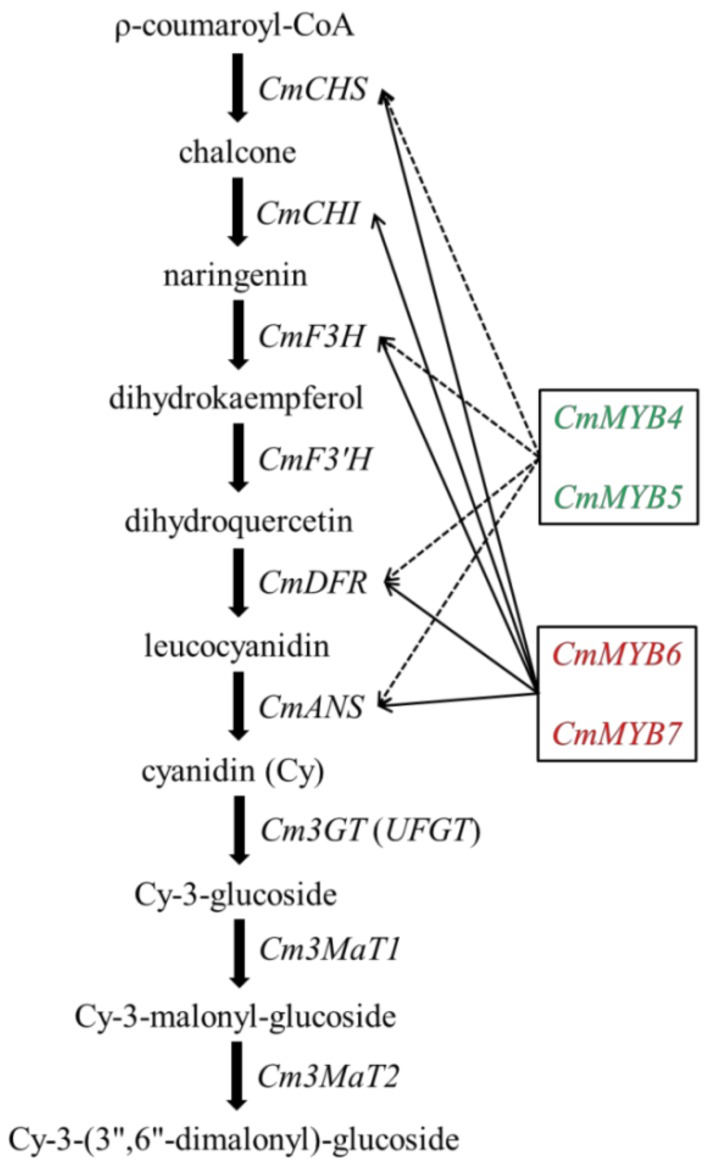
The inferred regulatory network of the anthocyanin biosynthetic pathway in the chrysanthemum. *CmMYB4* and *CmMYB5* negatively (marked by green with dashed line) regulate the expression of structural genes *CmCHS*, *CmF3H*, *CmDFR*, and *CmANS*, and correspondingly suppress the biosynthesis and accumulation of anthocyanins in the chrysanthemum capitulum. On the contrary, the expressions of *CmCHS*, *CmCHI*, *CmF3H*, *CmDFR*, and *CmANS* are positively regulated by *CmMYB6* and *CmMYB7* (marked by red with full line) and therefore promote the biosynthesis and accumulation of anthocyanins.

Previous studies have shown that among the three branches, only the cyanidin metabolic pathway exists in chrysanthemums [22,23]; therefore, *F3′H* is the key node gene for anthocyanin biosynthesis in the chrysanthemum, but not *F3′5′H*. To test the prediction that CmMYB4 and CmMYB5 are anthocyanin repressors while CmMYB6 and CmMYB7 are activators, as revealed by pigmental and phenotypic analyses, the ectopic expression of seven anthocyanin structural genes in tobacco was further examined by qRT-PCR. Reduced transcript levels were observed for four structural genes (*NtCHS*, *NtF3H*, *NtDFR*, and *NtANS*) in *35S:CmMYB4* and five (including the same four genes as for *35S:CmMYB4*) structural genes in *35S:CmMYB5* lines, while *NtCHS*, *NtCHI*, *NtF3H NtDFR*, and *NtANS* were all upregulated in both of the *35S:CmMYB6* and *35S:CmMYB7* lines, which partially supports the idea that CHS, CHI, F3H, DFR, and ANS might be coordinately controlled by multiple regulators in the chrysanthemum, whereas F3′H might be solely regulated by other TFs such as CmMYB5 (Figure 5).

Several mechanisms could be proposed to explain how CmMYBs act to reduce/increase transcription of these anthocyanin biosynthetic genes in tobacco. One possibility is that these regulators, acting similarly to the anthocyanin repressors AtMYB4 [54] and FaMYB1 [34] or anthocyanin activators PeMYB2/PeMYB12 (*Phalaenopsis* spp.) [55] and PpMYB9/PpMYB10.2 (*Pruns persica*) [56], recognize their respective normal target genes. In this case, CHS, CHI, F3H, DFR, ANS, and possibly F3′H might be the primary target sites. Another possibility could be that these four MYBs might non-specifically bind to target sites without the capability to repress/activate gene transcription and, therefore, may act by promoting/hindering the function of one or multiple tobacco regulator(s). Activation/repression might also be an indirect effect in which these CmMYBs non-specifically bind to other tobacco TFs that would be activated/inhibited in their function [18]. Finally, similar to CmMYB#7 [57], it is most likely that the four CmMYBs perform their regulatory functions by interacting with bHLH TF(s) and consequently activate or repress the expression of anthocyanin biosynthetic genes (see below).

### 4.3. The Combination of Protein–Protein Interactions among CmMYBs and bHLHs Needs to Be Further Studied

The combination of protein–protein interactions among R3-/R2R3-MYB and bHLH conservatively controls anthocyanin biosynthesis in many plants, such as PhAN2 (R2R3-MYB) and PhAN1 (bHLH) in petunia [58], Rosea1/Rosea2 (R2R3-MYB) and Delila (bHLH) in *Antirrhinum* spp. [10], GmMYB10 and GmMYC1 (bHLH) in *Gerbera* hybrids [59], as well as CmMYB#7 (R3-MYB) and CmbHLH2 in the chrysanthemum [57]. A key question that still needs to be directly answered for this study is whether these CmMYBs themselves can bind to promoters of anthocyanin biosynthetic genes and thus directly activate or repress anthocyanin gene expression, or, similar to the above studies, they perform their regulatory functions by interacting with bHLH TF(s).

Albert et al. suggested that R2R3-MYBs have the amino acid signature motif ([DE]Lx_2_[RK]x_3_Lx_6_Lx_3_R) [40], which has been identified as being important for interaction with R/B-like bHLH in Arabidopsis [60]. The fact that it is completely conserved within the MYB proteins that interact with R/B-like bHLH proteins not only from Arabidopsis but also from other species, including MYB proteins shown to act in the repression of transcription rather than activation, supports the view that a major function of this residue might be to stabilize interaction with bHLH proteins [60]. Additionally, it is worth noting that both MYB activators and repressors may simultaneously bind to the same bHLH cofactor(s) [7]. In *P. tomentosa*, a comparison of the bHLH-binding strength of MYB repressors and competing MYB activators using yeast two-hybrid assays found these to be comparable [61], suggesting that activation and repression at a promoter mainly depend on the relative abundance of MYB activator and repressor proteins in a given cell.

In this study, our data do not allow us to answer the question of whether the four CmMYB TFs perform their regulatory functions by interacting with bHLH cofactors, although the conserved motif [DE]Lx_2_[RK]x_3_Lx_6_Lx_3_R was found in all of their R3 domains. Moreover, whether these CmMYBs can bind to the same bHLH protein or not is still unknown. Investigation of protein–protein interactions among these CmMYBs, CmbHLH24 [26], and CmbHLH2 [57] using yeast two-hybrid and dual-luciferase assays is underway to answer these questions.

## Figures and Tables

**Table 1:** List of primers used in the present study.

Primer Name	Primer Sequence (3′–5′)	Purpose
*CmMYB4*-F	ATGGTGAGATCACCTTGTTGTG	Gene isolation
*CmMYB4*-R	TCATCTCCATCTACTCGACAAG	Gene isolation
*CmMYB5*-F	ATGACAAAACCTTGTTGTGATT	Gene isolation
*CmMYB5*-R	TTAGGGCACCACTGGTCCACTC	Gene isolation
*CmMYB6*-F	ATGAGACCGAGTAGTAGTACAG	Gene isolation
*CmMYB6*-R	TCATAGTTGGTCCGAATTTAAA	Gene isolation
*CmMYB7*-F	ATGGGAAGAACACCGTGTTGTG	Gene isolation
*CmMYB7*-R	TTAATTCCATTGCCAAAGAAAC	Gene isolation
*CmMYB4*-F1	CGGGATCCATGGTGAGATCACCTTGTTGTG	Vector construction
*CmMYB4*-R1	CGGAATTCTCATCTCCATCTACTCGACAAG	Vector construction
*CmMYB5*-F1	CGGGATCCATGACAAAACCTTGTTGTGATT	Vector construction
*CmMYB5*-R1	CGGAATTCTTAGGGCACCACTGGTCCACTC	Vector construction
*CmMYB6*-F1	CGGGATCCATGAGACCGAGTAGTAGTACAG	Vector construction
*CmMYB6*-R1	CGGAATTCTCATAGTTGGTCCGAATTTAAA	Vector construction
*CmMYB7*-F1	CGGGATCCATGGGAAGAACACCGTGTTGTG	Vector construction
*CmMYB7*-R1	CGGAATTCTTAATTCCATTGCCAAAGAAAC	Vector construction
*Ntα-Tub1*-F	ATGAGAGAGTGCATATCGAT	qRT-PCR analysis
*Ntα-Tub1*-R	TTCACTGAAGAAGGTGTTGAA	qRT-PCR analysis
*NtCHS*-F	TTGTTCGAGCTTGTCTCTGC	qRT-PCR analysis
*NtCHS*-R	AGCCCAGGAACATCTTTGAG	qRT-PCR analysis
*NtCHI*-F	GTCAGGCCATTGAAAAGCTC	qRT-PCR analysis
*NtCHI*-R	CTAATCGTCAATGCCCCAAC	qRT-PCR analysis
*NtF3H*-F	CAAGGCATGTGTGGATATGG	qRT-PCR analysis
*NtF3H*-R	TGTGTCGTTTCAGTCCAAGG	qRT-PCR analysis
*NtF3’H*-F	AGGCTCAACACTTCTCGT	qRT-PCR analysis
*NtF3’H*-R	CATCAACTTTGGGCTTCT	qRT-PCR analysis
*NtDFR*-F	TCCCATCATGCGATCAT	qRT-PCR analysis
*NtDFR*-R	ATGGCTTCTTTGTCACGTC	qRT-PCR analysis
*NtANS*-F	TGGCGTTGAAGCTCATACTG	qRT-PCR analysis
*NtANS*-R	GGAATTAGGCACACACTTTGC	qRT-PCR analysis
*Nt3GT*-F	GAGTGCATTGGATGCCTTTT	qRT-PCR analysis
*Nt3GT*-R	CCAGCTCCATTAGGTCCTTG	qRT-PCR analysis

## Supplementary Materials

The following are available online at https://www.mdpi.com/2073-4425/10/10/777/s1, Table S1: List of GenBank accession numbers of species used in the present study.
