# Supplementary material for: Ectopic Expression of Multiple Chrysanthemum (Chrysanthemum × morifolium) R2R3-MYB Transcription Factor Genes Regulates Anthocyanin Accumulation in Tobacco
Source: Genes (Basel). 2019 Oct 4;10(10):777. doi: 10.3390/genes10100777 (PMC6826627; doi:10.3390/genes10100777)
Supplement: Supplementary file 1 [file genes-10-00777-s001.pdf]

Supplementary Table S1. List of GenBank accession numbers of species used in the present study.

Gene Name	GenBank Accession Number	Gene Name	GenBank Accession Number
AtMYB0	AT3G27920.1	AtMYB1	AT3G09230.1
AtMYB2	AT2G47190.1	AtMYB3	AT4G01060.1
AtPC-MYB1	AT4G32730.2	AtMYB3R-2	AT4G00540.1
AtMYB3R-3	AT3G09370.2	AtMYB3R-4	AT5G11510.1
AtMYB3R-5	AT5G02320.1	AtMYB6	AT4G09460.1
AtMYB7	AT2G16720.1	AtMYB8	AT1G35515.1
AtMYB9	AT5G16770.1	AtMYB10	AT3G12820.1
AtMYB11	AT3G62610.1	AtMYB12	AT2G47460.1
AtMYB13	AT1G06180.1	AtMYB14	AT2G31180.1
AtMYB15	AT3G23250.1	AtMYB16	AT5G15310.1
AtMYB17	AT3G61250.1	AtMYB18	AT4G25560.1
AtMYB19	AT5G52260.1	AtMYB20	AT1G66230.1
AtMYB21	AT3G27810.1	AtMYB22	AT5G40430.1
AtMYB23	AT5G40330.1	AtMYB24	AT5G40350.1
AtMYB25	AT2G39880.1	AtMYB26	AT3G13890.1
AtMYB27	AT3G53200.1	AtMYB28	AT5G61420.2
AtMYB29	AT5G07690.1	AtMYB30	AT3G28910.1
AtMYB31	AT1G74650.1	AtMYB32	AT4G34990.1
AtMYB33	AT5G06100.2	AtMYB34	AT5G60890.1
AtMYB35	AT3G28470.1	AtMYB36	AT5G57620.1
AtMYB37	AT5G23000.1	AtMYB38	AT2G36890.1
AtMYB39	AT4G17785.1	AtMYB40	AT5G14340.1
AtMYB41	AT4G28110.1	AtMYB42	AT4G12350.1
AtMYB43	AT5G16600.1	AtMYB44	AT5G67300.1
AtMYB45	AT3G48920.1	AtMYB46	AT5G12870.1
AtMYB47	AT1G18710.1	AtMYB48	AT3G46130.1
AtMYB49	AT5G54230.1	AtMYB50	AT1G57560.1
AtMYB51	AT1G18570.1	AtMYB52	AT1G17950.1
AtMYB53	AT5G65230.1	AtMYB54	AT1G73410.1
AtMYB55	AT4G01680.2	AtMYB56	AT5G17800.1
AtMYB57	AT3G01530.1	AtMYB58	AT1G16490.1
AtMYB59	AT5G59780.3	AtMYB60	AT1G08810.1
AtMYB61	AT1G09540.1	AtMYB62	AT1G68320.1
AtMYB63	AT1G79180.1	AtMYB64	AT5G11050.1
AtMYB65	AT3G11440.1	AtMYB66	AT5G14750.1
AtMYB67	AT3G12720.1	AtMYB68	AT5G65790.1
AtMYB69	AT4G33450.1	AtMYB70	AT2G23290.1
AtMYB305	AT3G24310.1	AtMYB72	AT1G56160.1
AtMYB73	AT4G37260.1	AtMYB74	AT4G05100.1

Gene Name	GenBank Accession Number	Gene Name	GenBank Accession Number
AtMYB75	AT1G56650.1	AtMYB76	AT5G07700.1
AtMYB77	AT3G50060.1	AtMYB78	AT5G49620.2
AtMYB79	AT4G13480.1	AtMYB80	AT5G56110.1
AtMYB81	AT2G26960.1	AtMYB82	AT5G52600.1
AtMYB83	AT3G08500.1	AtMYB84	AT3G49690.1
AtMYB85	AT4G22680.1	AtMYB86	AT5G26660.1
AtMYB87	AT4G37780.1	AtMYB88	AT2G02820.2
AtMYB89	AT5G39700.1	AtMYB90	AT1G66390.1
AtMYB91	AT2G37630.1	AtMYB92	AT5G10280.1
AtMYB93	AT1G34670.1	AtMYB94	AT3G47600.1
AtMYB95	AT1G74430.1	AtMYB96	AT5G62470.2
AtMYB97	AT4G26930.1	AtMYB98	AT4G18770.1
AtMYB99	AT5G62320.1	AtMYB100	AT2G25230.1
AtMYB101	AT2G32460.1	AtMYB102	AT4G21440.1
AtMYB103	AT1G63910.1	AtMYB104	AT2G26950.1
AtMYB105	AT1G69560.1	AtMYB106	AT3G01140.1
AtMYB107	AT3G02940.1	AtMYB108	AT3G06490.1
AtMYB109	AT3G55730.1	AtMYB110	AT3G29020.2
AtMYB111	AT5G49330.1	AtMYB112	AT1G48000.1
AtMYB113	AT1G66370.1	AtMYB114	AT1G66380.1
AtMYB115	AT5G40360.1	AtMYB116	AT1G25340.1
AtMYB117	AT1G26780.2	AtMYB118	AT3G27785.1
AtMYB119	AT5G58850.1	AtMYB120	AT5G55020.1
AtMYB121	AT3G30210.1	AtMYB122	AT1G74080.1
AtMYB123	AT5G35550.1	AtMYB124	AT1G14350.1
AtMYBC1	AT2G40970.1	AmROSEA1	ABB83826.1
AmROSEA2	ABB83827.1	AmVENOSA	ABB83828.1
AmMYB308	P81393.1	CsRuby	NM_001288889
FaMYB1	AF401220	FaMYB9	JQ989281
FaMYB11	JQ989282	LjTT2a	AB300033
MdMYB10a	ABB84754.1	MdMYB22	AAZ20438.1
OsMYB3	BAA23339.1	PhAN2	AAF66727.1
PtMYB182	AJI76863.1	TaMYB14	AFJ53053.1
VvMYBA1	BAD18977.1	VvMYBC2-L1	AFX64995.1
VvMYBF1	ACV81697.1	VvMYBPA2	ACK56131.1
PhMYB27	AHX24372.1	MdMYB16	NP_001315806.1
ZjMYB12	XP_015891841.1	MdMYB1	ABK58136.1
PhPHZ	ADW94951.1	PhDPL	ADW94950.1
